# Potential Triggers for Risking the Development of Eating Disorders in Non-Clinical Higher-Education Students in Emerging Adulthood

**DOI:** 10.3390/nu14112293

**Published:** 2022-05-30

**Authors:** Marius Baranauskas, Ingrida Kupčiūnaitė, Rimantas Stukas

**Affiliations:** 1 Faculty of Biomedical Sciences, Panevėžys University of Applied Sciences, 35200 Panevėžys, Lithuania; ingrida.kupciunaite@panko.lt; 2 Department of Public Health, Institute of Health Sciences, Faculty of Medicine, Vilnius University, 01513 Vilnius, Lithuania; rimantas.stukas@mf.vu.lt

**Keywords:** eating disorder, higher-education students, mental health, emerging adulthood, COVID-19 pandemic

## Abstract

Nowadays, eating disorders (ED) among individuals during emerging adulthood have become a crucial challenge to public health, taking into account the fact that the global prevalence of the ED risk in student-aged populations already stands at 10.4% and has been sharply increasing during the COVID-19 pandemic. In all, from 50% to 80% of all the ED cases go undetected or are not correctly diagnosed; moreover, these individuals do not receive specialized treatment. Therefore, early diagnosis detected via screening questionnaires for ED is highly recommended. This study aimed to identify the triggers for ED risk development in emerging-adulthood individuals and to reveal the factors significant not only for ED prevention but also for assessing individuals with subthreshold symptoms. This cross-sectional study provides the results for the ED symptom screening in 1716 Lithuanian higher-education students aged 21.2 ± 3.9, during emerging adulthood. According to the results of this study, 19.2% of students were at risk for ED. Potential risk factors such as sex (odds ratio (OR): 3.1, 95% CI: 1.9–4.9), body weight (self-reported body mass index) (adjusted (A) OR: 1.4; 95% CI: 1.2–1.7) and comorbidities such as smoking (AOR: 2.1; 95% CI: 1.6–2.8), and perceived stress during the pandemic (AOR: 1.4; 95% CI: 1.1–1.8) are involved in anticipating the symptomatology of ED during emerging adulthood. Regular initial screenings with universally adopted questionnaires and further referral to a psychiatrist must be applied to promote both the diagnosis of early-onset symptomatology and the treatment of these ED in student-aged populations. Preventive programs for reducing the prevalence of overweight or obesity among students during emerging adulthood should focus on integration directions for the development of a positive body image.

## 1. Introduction

Eating disorders (ED) are a group of serious psychiatric disorders that are further classified into anorexia nervosa (AN), bulimia nervosa (BN), binge eating disorder (BED), unspecified feeding or eating disorder (UFED) and those characterized by both abnormal eating behaviour and distorted body image [1]. Epidemiological surveys have indicated a lifetime prevalence of DSM-5 defined BED, AN, and BN of 1.53%, 0.16%, and 0.63%, respectively [2].

According to global scientific forecasts, 15% of women and 4% of men will be affected by ED at some point in their lives [2,3,4,5]. In the meantime, it is undoubtedly important to focus on the prevalence of ED during the more specific age period identified as emerging adulthood, when an individual (aged~18–29) performs adult roles after graduating from high school [6]. During this stage of emerging adulthood, the occurrence of mental health diseases [7], including ED, has also been observed as part of outcomes triggered by the physical, psychological and social development of persons [8,9,10,11], and has been associated with alterations in brain structures and functions during maturation [12].

Students fall into one of the highest risk groups depending on their financial burden, poor eating habits, social support, and the abnormal psychological stress associated with academic strain during EA [13,14,15,16]. The academic year is filled with not only high levels of perceived stress, but also with weight-related concerns, a negative body image, and disturbed eating habits, especially among women. On the other hand, it is consistent with the occurrence and development of ED in student-aged populations. On that account, EDs among current higher-education students in emerging adulthood have become an important public health challenge [4], especially when the global pre-pandemic prevalence of ED risk in the student population was identified at 10.4% [17], which has been sharply increasing during the COVID-19 pandemic [18].

Taking into account that lower academic functioning [19], somatization, comorbid mental disorders [20,21], increased suicide risk [22] were identified as the main deleterious outcomes of ED in emerging adulthood individuals, it can be claimed that the ED morbidity of students attending higher-education institutions undoubtedly triggers unfavourable consequences in both individuals and societies. Among mental health disorders, ED were also associated with the highest rates of disability and mortality [23,24,25,26,27]. Due to a lack of awareness or a lack of a sufficiently defined method for diagnosing ED, from 50% to 80% of all cases go undetected or are not correctly diagnosed. As a result, the majority of ED patients do not have access to evidence-based effective treatments [27,28,29,30,31,32]. Early diagnosis using questionnaires for ED screening is highly recommended [33,34]. Another important reason why both early prevention and psychosocial interventions for the population potentially suffering from ED to date have not been effective is the poor knowledge status of the mechanistic processes leading to the ED risk and full-blown ED [35,36,37,38]. The risk factors and comorbidities associated with ED have not been also sufficiently identified among emerging adults with ED [37]. Nevertheless, there are several factors highlighted in the scientific literature that could be related to ED in emerging adulthood individuals, namely, age, sex, marital status, greater exposure to western culture, cultural transition and health-related variables, including body mass index [39,40,41,42,43,44]. This study aimed to identify the triggers for the development of ED risk in emerging adulthood individuals and to reveal the factors facilitating ED prevention that are considered in assessing an individual with specific subthreshold symptoms. We identified the following challenges: (1) the primary aim of this study was to assess the ED risk in emerging-adulthood individuals as well as to reveal the association between potential risk factors, comorbidities of ED and increased/decreased ED symptomatology; (2) considering that both ED symptomatology and behaviours related to ED can play a significant role in triggering changes in body mass index (BMI) [18,45,46,47], the secondary aim of the study was to identify the association between ED symptomatology, behaviours related to ED and the self-reported BMI of subjects.

## 2. Materials and Methods

### 2.1. Study Population, Area and Design

This observational study provided the screening of ED risk fifteen months after the start of the COVID-19 pandemic declared by WHO on 11 March 2020 [48]. A cross-sectional study was carried out among students of higher-educational institutions from September to December 2021. According to Kelsey’s formula [49], we calculated the representative sample size for 1702 cases of the population by considering a 4% (z-score 1.96) margin of error, 95% two-sided confidence interval (CI), and 20.8% overall prevalence of the risk for ED in students of higher-education institutions [18].

We used a simple random sampling survey design, in which 11 Lithuanian universities and 10 higher-education colleges were considered as clusters. A total of 84 faculties of higher-education institutions with the 1st–6th year bachelor, master, doctoral and residency degree students were selected from 10 cities (Vilnius, Kaunas, Klaipėda, Šiauliai, Panevėžys, Telšiai, Utena, Tauragė, Marijampolė, Alytus) in Lithuania.

The recruitment procedure of students of higher-education institutions followed the method of convenience sampling. The web-based E-survey research application was used to collect information (https://apklausa.lt/private/forms/ (accessed on 6 September 2021)). In September 2021, higher-education-institution-wide email distribution lists were used to invite students to participate in the non-experimental cross-sectional study. Provided the students were interested in participating in the survey, they were asked to follow a link to the website with an online questionnaire. An e-survey link was also distributed through social media.

The number of students who answered the e-survey was 1747. The inclusion criteria were set out in the following terms: those currently studying at a higher-education institution, aged 18 years or more, a resident of the Republic of Lithuania with negative pregnancy status, the ability to read and understand Lithuanian, and consent to respond the survey questionnaire. The responses of participants over 29 years old or those who did not complete or respond to online questionnaires properly were excluded. In addition, after identifying duplicate replies (by checking on age, sex, academic course, branch of science, geographic location where the electronic device such as computer, or smartphone was used, the time when the questionnaires were completed and specific time limits for submitting the completed questionnaire), these were removed. The final analysis of the study sample included 1716 respondents of the age range from ≥18 to ≤29 (mean age was 21.2 ± 3.9 years).

### 2.2. Measures

#### 2.2.1. Symptomatology and Risk of ED

Eating attitudes test (EAT-26), as a valid tool [50,51] created by Garner et al. [52], was used in a two-stage screening as the first step to determine the risks of abnormal eating behaviours in a student population in Lithuania. Depending on the type of responses provided to the 26 questions of the test, the total score could range from 0 to 78. The ED symptomatology was defined as participant score on EAT-26. A cut-off score of 20 was designed to find out the presence or the absence of the ED risk (or to determine the ED cases from no-cases) [52,53]. If an increased ED symptomatology was detected, it was recommended to visit a mental health professional. The EAT-26 was divided into three subsections B (bulimia), D (dieting), O (oral control) and allowed for more information from the same questionnaire, further classified into Factor D (dieting) (13 items), which provided a tendency towards a distorted body image; Factor B (bulimia) (6 items), which was closely related to a bulimic behaviour; and Factor O (oral control) (7 items), which was correlated with a low body weight without bulimia. EAT-26 allowed us to identify the dietary behaviours associated with ED (5 additional items), which were classified into binge eating, self-induced vomiting, the use of laxatives, diuretics, diet pills, problematic use of physical activity (>60 min a day) in order to control body weight and extreme weight loss (9 kg or > in the past 6 months).

#### 2.2.2. Potential Risk Factors and Comorbidities of ED

The study participants answered a questionnaire concerning their socio-demographic characteristics (age; type of branch of science: medicine and health sciences, natural sciences, social sciences, humanities, agricultural sciences, technological sciences, or arts) and personal data related to the potential ED risk factors such as sex; income (EUR per month) and body-weight status. According to the recommendations for measuring body mass and height presented in the questionnaire, the subjects submitted estimates of body mass (in kg) and height (in cm). Body mass index (BMI, kg/m^2^) was calculated from self-reported data by dividing the weight (kg) by the height (m) squared. According to the BMI classification categories recommended by the World Health Organization (WHO), the subjects were divided by weight status into four groups (underweight: BMI < 18.5 kg/m^2^, normal weight: BMI 18.5 to < 25 kg/m^2^ (reference category), overweight: BMI 25.0 to < 30 kg/m^2^ and obesity: BMI ≥ 30 kg/m^2^) [54].

Data were collected on the questionnaire by additionally integrating issues related to the comorbidities of ED such as alcohol consumption (yes/no), drug use (yes/no), cigarette-smoking status (yes/no), and including variables related to the pandemic of the coronavirus disease (COVID-19), further split into 3 items about COVID-19 cases (yes/no), pandemic-dependent self-isolation (yes/no) and psychologic distress (yes/no).

### 2.3. Statistical Analysis

The statistical analysis was performed using SPSS V.25 for Windows (Armonk, NY, USA) and Microsoft Excel (Seattle, WA, USA). The normality of variable distribution was tested by the Shapiro–Wilk *W*-test. All normally distributed continuous variables were calculated as means ± standard deviations (SD), whereas categorical variables were presented as relative frequencies (by percentage). The differences in EAT-26 score (20 > EAT-26 score ≥ 20) between groups of each covariate were assessed using Pearson’s χ^2^ test, and using Cramer’s V (V) and phi (φ) correlation coefficients. A higher degree of the absolute value of the coefficient described a stronger relationship between the variables in contingency tables. The correlations above 0.4 were considered to be relatively strong and strong; the correlations between 0.2 and 0.4 were moderate, and those below 0.2 were weak. The critical level of the significance was considered α = 0.05.

To address the first aim, the multivariate logistic regression model was fit to test the association between the increased/decreased ED symptomatology (dependent variable) and its potential risk factors, with comorbidities (self-reported BMI, smoking status, alcohol abuse, psychological distress during the COVID-19 pandemic) as the independent variables. The stepwise multivariate logistic regression method was used to establish which potential risk factors and comorbidities were related to EAT-26 score < 20 and EAT-26 score ≥ 20.

Based on the fact that BMI can serve as the dependent variable in relation to ED symptomatology and behaviours related to ED [18,45,46,47], to address the second aim, the multivariate logistic regression model was fit to test the association between the self-reported BMI of subjects and the independent variables such as ED symptomatology (20 > EAT-26 score ≥ 20: 2.99 > Factor D (dieting) score ≥ 3, 0.99 > factor B (bulimia) score ≥ 1, 1.99 > Dactor O (oral control) score ≥ 2) and eating behaviours related to ED (BE, problematic use of physical activity in order to lose or to control weight, self-induced vomiting, the use of laxatives, diuretics or diet pills, the weight loss practices). The stepwise multivariate logistic regression method was used to establish which specificities and levels of ED symptomatology, as well as what kind of eating behaviours related to ED, were associated with the body-weight status of subjects (underweight: self-reported BMI < 18.5 kg/m^2^, normal weight: self-reported 18.5 kg/m^2^ ≥ self-reported BMI ≤ 24.9 kg/m^2^ (reference category), overweight and obesity: self-reported BMI ≥ 25 kg/m^2^). Logistic regression models were adjusted for sex and branch of sciences. Goodness-of-fit of logistic regression models was assessed using the Wald test and Nagelkerke R^2^ statistic.

## 3. Results

### 3.1. The ED Symptomatology, Potential Risk Factors and Comorbidities

Among 1716 students, 330 (19.2%) were at risk for ED. The cohort composition for the ED risk by a branch of science, sex, BMI, income, drug and alcohol abuse, tobacco use and psychological distress during the COVID-19 pandemic is presented in Table 1. A higher risk of ED was observed among female than male subjects (21.4% vs. 6.9%, OR: 3.1, 95% CI: 1.9–4.9, *p* < 0.0001).

The results of the study showed a positive weak correlation between a higher risk of ED and variables such as a branch of science (V = 0.13, *p* < 0.0001), self-reported BMI (V = 0.14, *p* < 0.0001), alcohol consumption (φ = 0.06, *p* = 0.02), smoking status (φ = 0.15, *p* < 0.0001) and psychological stress due to the COVID-19 pandemic (φ = 0.06, *p* = 0.01).

The results of the multivariate analysis are displayed in Table 2. Multivariate logistic regressions were constructed to obtain how self-reported BMI, tobacco use, alcohol abuse and the perceived stress during the COVID-19 pandemic can associate with the risk of ED (EAT-26 score ≥ 20). The potential risk factors and comorbidities such as self-reported BMI (AOR: 1.4; 95% CI: 1.2–1.7, *p* < 0.0001), smoking status (Adjusted OR (AOR): 2.1; 95% CI: 1.6–2.8, *p* < 0.0001), and the perceived stress during the COVID-19 pandemic (AOR: 1.4; 95% CI: 1.1–1.8, *p* = 0.017) were associated with a score at or above 20 on the EAT-26 and a higher risk of ED.

### 3.2. Association between the ED Symptomology, Eating Behaviour and the Weight Status

Among this sample of respondents, 17% were underweight, 21.7% were overweight and 4.7% were obese. Compared to the underweight and normal-weight subsamples (14.4% and 16.9%), there were more students at risk of ED in the overweight and obese group (27.4%) (*p <* 0.0001).

The ED risk was more frequently associated with specific behaviours such as BE (59%) and problematic use of physical activity in order to lose or control weight (41.4%) in a sample of students. Less frequently, the subjects also practiced behaviours considered to be self-induced vomiting (11.2%), the use of laxatives, diuretics or diet pills (7.7%) and weight-loss practices (10.3%). The key characteristics of eating behaviour in the past 6 months according to the category of the ED risk are provided in Table 3.

Table 4 displays a multivariate logistic regression analysis of the model. The model showed the association between the EAT-26 scale with its subscales (Factor: D (dieting), B (bulimia), O (oral control)), behaviours associated with ED and the self-reported body-weight status (underweight, overweight and obesity) of students. The regression of the model identified that overweight and obesity in students were associated with higher EAT-26 dieting subscale (D) scores related to a distorted body image (AOR: 3.4; 95% CI: 2.5–4.7, *p* < 0.0001), eating behaviour such as BE (AOR: 1.1; 95% CI: 1–1.3, *p* = 0.027) and a problematic use of physical activity in order to control body weight (AOR: 1.1; 95% CI: 1–1.2, *p* = 0.017). Meanwhile, an underweight status of subjects was associated with the EAT-26 oral control (O) subscale scores related to low body weight and the absence of BN (AOR: 4.8; 95% CI: 3.4–6.8, *p* < 0.0001), eating behaviour such as self-induced vomiting (AOR: 1.3; 95% CI: 1–1.6, *p* = 0.035) and the use of laxatives, diuretics or diet pills (AOR: 1.3; 95% CI = 1–1.7, *p* = 0.036).

## 4. Discussion

### 4.1. ED Symptomatology and Disordered Eating Behaviours in Emerging Adulthood Individuals

Taking into account the meta-analysis study data published by other researchers (Jahrami H et al. [17], Potterton R et al. [55]) the global prevalence of ED risk among students was estimated at 10.4%, especially amongst emerging adults. Although the prevalence of ED was stable between the period of 2009 and 2018, it increased dramatically from the start of the COVID-19 pandemic [18]. According to our study, 19.2% of Lithuanian higher-education students during emerging adulthood were at risk for ED. The results of the similar positive pre-pandemic ED screenings (from 16% to 33%) can be observed in the student populations in Nigeria [56], Lebanon [57], Singapore [58], Spain [59], Palestine [60], Pakistan [61], Turkey [62], Bangladesh [63], Iran [64,65], the United Arab Emirates [66], and Egypt [67]. Meanwhile, significantly lower pre-pandemic reports of the prevalence of the ED risk among students ranging from 4.5% to 14.9% were identified in the countries such as Poland [68], Croatia [69], the United States [70,71], Puerto Rico [72], Brazil [73], China [74,75], Japan [76], South India [29], and Malaysia [77].

According to our study results, the ED symptoms appeared in conjunction with BE and “over exercise” in order to lose or to control weight. Less frequently, Lithuanian students also practiced purging and non-purging behaviours [78] considered to be self-induced vomiting and the use of laxatives, diuretics or diet pills and weight-control practices. Similar results were published by other authors claiming that approximately a quarter of students practiced unhealthy weight-control methods such as self-induced vomiting, fasting, excessive exercise, or taking laxatives or diuretics at least once per week [79]. Meanwhile, one in ten students took part in BE [79,80,81,82].

### 4.2. Potential Risk Factors for the ED Development

The most common factors associated with ED are age, marital status, sex, a greater impact of Western culture, psychological stress, and health-related variables including BMI [39,40,41,83]. According to our study, a 3.1-times higher risk of ED was found among female compared to male subjects. The findings of our study coincide with other traditional empirical data confirming a higher risk for developing ED among women [43,84,85]. However, due to large disproportions between the comparison groups of female and male subjects, in spite of the use of appropriate tests, the observed ED symptomatology depending on sex differences should be approached with caution. Additionally, the findings supported by other studies identified an increased ED risk among homosexual men during emerging adulthood [43,86,87,88]. Thus, further research is needed to clarify the relationship between abnormal eating behaviours and a higher body dissatisfaction in a sample of homosexual men students during emerging adulthood.

Another interesting finding obtained from our study was related to self-reported weight status in the student group with the ED symptoms. There is evidence that BMI can be considered a significant predictor of the outcome in AN [89]. In connection with this, our study identified that a higher EAT-26 oral control (O) subscale score was related to a higher prevalence of underweight students and abnormal eating behaviour such as self-induced vomiting and the use of laxatives, diuretics or diet pills. Furthermore, based on scientific data, the ED that have been most frequently observed in subjects with obesity were BN and BE, both of which are characterized by abnormal eating or weight-control behaviours [90,91]. On the contrary, we identified the absence of BN, but the association between a distorted body image, behaviours such as BE, and a higher risk for overweight or obesity in students was revealed. Thus, a distorted body image may be an important predictor for being overweight. According to our study, abnormal self-reported BMI values (overweight and obesity) were also related to the problematic use of physical activity among students suffering from ED symptoms. Interestingly, abnormally high levels of physical activity were identified as a risk factor only in the earliest clinical description of AN as an ED [92,93,94]. At present, there is still no data available for other ED symptoms. There is no consensus on how to define, conceptualize or prevent the observed high levels of physical activity in underweight and obese individuals suffering from ED. We hypothesized that, in order to maximize weight loss, an excess of physical activity is initially considered as a conscious action. However, as weight loss improves, the levels of physical activity also proportionally increases, which can potentially be controlled by a developed unconscious biological drive and involuntary cognition [94].

Our study also reported that 16.9% of students with normal weight still suffered from ED according to EAT-26. In this case, the development of ED may be explained from a psychological standpoint and even subjects with a normal BMI may have an increased risk of the ED symptomatology [61,63].

### 4.3. Comorbidities of ED

Many individuals do not experience ED in isolation and can suffer from many psychological and non-psychological comorbidities. Psychological comorbidities associated with ED occur as mental health disorders such as depression, bipolar disorder [1], anxiety [95,96], panic disorder [71], and social phobia [97]. In Lithuania, our study showed that students, due to the perceived psychological distress during the COVID-19 pandemic, were more likely to screen positive for an ED. These results were in line with the literature that concluded that there are a variety of stressors that may contribute to increased levels of psychological stress, anxiety, and depressive thoughts as students live through the COVID-19 pandemic [98]. In the meantime, psychological stress could correlate with the symptomatology of mental health disorders and ED and mediate both outcomes specifically [44,79,81,99,100,101,102,103,104]. The association between the psychological distress during the COVID-19 pandemic and the increased risk for ED symptoms in students can also be explained by the interaction between emotional distress and coping behaviours such as eating a poor diet and comfort food, adopted as a consequence of the COVID-19-related stressors [83,105,106] and a lack or modified paid activity [107], which determines food insecurity [108,109].

In addition to psychological comorbidities, individuals with ED (AN, BN, BED) often suffer from substance, such as alcohol and drug, abuse or addiction/dependence [1,20,97,110,111,112,113]. Based on our study data, unlike alcohol consumption, smoking status as a comorbidity was associated with higher levels of ED risk. In line with this interesting finding of our study, it should be taken into account that, earlier, it had also been established that people with BED and BN (in the absence of AN) had tended to be life-time smokers [114]. Meanwhile, other authors [115] published minimal differences in the ED psychopathology by smoking status in adults. However, very little is known about cigarette smoking rates in accordance with ED risk during emerging adulthood. At present, scientific knowledge only reveals that a malnourished population with AN could have an emphysema-like changes in lungs, while smoking may induce lung damage, which could be related to a higher risk for lung cancer [116,117]. Meanwhile, cigarette use has been associated with a higher likelihood of cardiovascular disease development in obese individuals diagnosed with BED [118,119]. Thus, determining the prevalence of smoking among emerging adulthoods who are diagnosed with ED during the COVID-19 pandemic and comparing it with the general population could be an important public health issue.

### 4.4. Strengths and Limitations of the Study

The main strengths of this study were: (1) a large sample size which made it possible to generalize the results for the entire population of Lithuanian higher-education students during emerging adulthood; and (2) an objective screening of the ED symptomatology using a validated instrument. However, this study had some limitations. First of all, our study was cross-sectional in design, limiting the conclusions that can be formed about causality. Secondly, caution should be applied when assessing the relationship between BMI, physical-activity profile and ED symptomatology. In population studies, BMI is usually estimated due to self-reported body weight and height. Nonetheless, the self-reporting of anthropometrics can be biased according to chronic conditions such as diabetes, cardiopathy, hepatopathy, gastric ulcer, cancer, bronchial asthma, hypertension, hyperlipidemia, cerebral apoplexy, and osteoporosis, interfering BMI values. For a more accurate assessment of this association, it would be optimal to carry out additional measurements of body composition (body weight, lean body mass, muscle mass, body fat) using bioelectrical impedance analysis (BIA) [120] or dual-energy X-ray absorptiometry (DXA) [121]. When performing measurements of physical activity levels, the intensity of physical activity and the characteristics (in terms of aerobic and anaerobic) developed by athletes must be also taken into account. Lastly, notwithstanding the fact that a lifetime prevalence of DSM-5 defined BN was two–three times lower than the prevalence in the BED sample of the general population [2], the results of our study based on the EAT-26 questionnaire as a screening tool were limited concerning bulimic behaviour [122]. Therefore, future empirical studies on the student population during emerging adulthood should be carried out using a couple of instruments such as complementary questionnaires (e.g., the scales of both EAT-26 [52] and SCOFF tests [123]). Further cross-sectional studies, including longitudinal studies in design, should focus on the observation and identification of which positives for ED screening correspond to actual ED diagnoses and their dynamics in emerging adulthoods during the COVID-19 pandemic. Further research is also needed to reveal the potential association between the development of ED risk in a student population and the use of comorbid substances such as nicotine, caffeine and stimulants that can be appetite suppressants.

## 5. Conclusions

The increased risk for developing eating disorders is typical of almost one in five of higher-education students. Potential risk factors such as sex, self-reported body weight and comorbidities, namely, smoking or perceived stress during the COVID-19 pandemic, may trigger the eating disorder symptomatology during emerging adulthood. Nevertheless, almost 17% of individuals with normal weight are still suffering from eating disorder symptoms. It can be concluded that the body-weight status of emerging adulthoods plays an important role in mediating the association between body mass index and the behaviours related to eating disorders, rather than indirectly maintaining the risk of eating disorder psychopathology. These findings are supported by evidence from this study: that the lowest prevalence of eating disorder symptoms persists among underweight students with abnormal eating behaviours such as self-induced vomiting and the use of laxatives, diuretics or diet pills. Meanwhile, the majority of overweight and obese students have positive eating disorder screenings in relation with a distorted body image and behaviours such as binge eating and a problematic use of physical activity in order to control body weight.

Several eating-disorder triggers intertwine and occur together with a higher likelihood of indicating abnormal eating behaviours in a more specific way in the student population during emerging adulthood, thus opening up the opportunity of translating these revelations into a form of intervention. Regular initial screening with universally adopted questionnaires and further referral to a psychiatrist must be applied to promote both the diagnosis of early-onset symptomology and treat these eating disorders in the student-aged population. Preventive, clinical and higher-education school-based intervention programs in reducing the prevalence of overweight or obesity among students during emerging adulthood should focus on integration directions for positive body-image development.

## Figures and Tables

**Table 1:** Student distribution according to the ED risk category.

Variables	EAT-26 Score		Total (N = 1716)	V ^†^/φ *	*p*
	No > Low-Risk ED ^1^ (N = 1386)	High-Risk ED ^2^ (N = 330)			
Branch of science ^†^				0.13	<0.0001
Medicine and Health Sciences (%)	82.2	17.8	40.3		
Natural Sciences (%)	89.4	10.6	7.7		
Social Sciences (%)	78.2	21.8	30.2		
Humanities (%)	69	31	6.6		
Agricultural Sciences (%)	68.4	31.6	2.2		
Technological Sciences (%)	86.7	13.3	9.6		
Arts (%)	81	19	3.4		
Sex *				−0.13	<0.0001
Female (%)	78.6	21.4	84.8		
Male (%)	93.1	6.9	15.2		
Body weight (BMI) (kg/m^2^) ^†^				0.14	<0.0001
Underweight (<18.5) (%)	85.6	14.4	17		
Normal weight (18.5–24.9) (%)	83.1	16.9	56.6		
Overweight (25.0–29.9) (%)	74.5	25.5	21.7		
Obese (30.0–34.9) (%)	64.2	35.8	4.7		
Income (euros (EUR) per month) ^†^				0.05	0.12
<200 (%)	83.7	16.3	32.5		
200–500 (%)	79.4	20.6	42.7		
>500 (%)	79.3	20.7	24.8		
Drug abuse (positive) (%) *	76.5	23.5	6.9	0.03	0.22
(negative) (%)	81.1	18.9	93.4		
Alcohol abuse (positive) (%) *	79.1	20.9	64	0.06	0.02
(negative) (%)	83.7	16.3	36		
Smoking status (positive) (%) *	73.5	26.5	38.3	0.15	<0.0001
(negative) (%)	85.3	14.7	61.7		
COVID-19 case (positive) (%) *	78.8	21.2	20.6	0.03	0.29
(negative) (%)	81.3	18.7	79.4		
Distress during the COVID-19 pandemic (positive) (%) *	78.1	21.9	45	0.06	0.01
(negative) (%)	82.9	17.1	55		
Self-isolation because of COVID-19 (positive) (%) *	79.1	20.9	36.2	0.03	0.18
(negative) (%)	81.7	19.2	63.8		

* φ—the Phi correlation coefficient; ^†^ V—the Cramer’s V correlation coefficient; *p*—*p* value; ^1^—EAT-26 score < 20, ^2^—EAT-26 score ≥ 20; yr.—year; BMI—body mass index; COVID-19—coronavirus disease.

**Table 2:** The association between the potential risk factors and comorbidities such as current body weight (self-reported BMI), smoking status, psychological distress during the COVID-19 pandemic and the risk of ED (EAT-26 score ≥ 20/EAT-26 score < 20).

High-Risk of ED (EAT-26 Score ≥20) ^a^ (Nagelkerke R^2^ = 0.26)	β	W	*p*	AOR (95% CI)
Smoking status	0.7	24.7	<0.0001	2.1 (1.6, 2.8)
Alcohol abuse	0.1	0.1	0.873	1.1 (0.8, 1.4)
Perceived stress during the COVID-19 pandemic	0.3	5.7	0.017	1.4 (1.1, 1.8)
Body weight status (BMI) (kg/m^2^)	0.4	16.1	<0.0001	1.4 (1.2, 1.7)

^a^—reference category is no to low-risk of ED (EAT-26 score < 20); β—the estimated coefficient; W—the Wald test statistic; CI—confidence interval; AOR—adjusted odds ratio; *p*—*p*-value; multivariate logistic regression model was adjusted for sex and branch of sciences.

**Table 3:** Student distribution by eating behaviour according to the ED risk category.

Eating Behaviour in the Past 6 Months	Once or Less a Month	2–3 Times a Month	Once a Week	2–6 Times a Week	Once a Day	V ^†^/φ *	*p*
Binge eating ^†^ (total) (%)	32.1	14.5	5.7	5.5	1.3	0.43	<0.0001
No > Low-Risk ED ^1^ (%)	34	12.9	4.1	2.3	0.1		
High-Risk ED ^2^ (%)	24.2	21.2	12.1	18.8	6.4		
Self-induced vomiting ^†^ (total) (%)	6.9	1.9	0.6	1.3	0.5	0.41	<0.0001
No > Low-Risk ED ^1^ (%)	4	0.7	0.1	0.4	0		
High-Risk ED ^2^ (%)	19.1	6.7	2.7	4.8	2.7		
Laxatives, diet pills, diuretics ^†^ (total)	5.1	1	0.7	0.7	0.2	0.29	<0.0001
No > Low-Risk ED ^1^ (%)	3	0.4	0.2	0.2	0.1		
High-Risk ED ^2^ (%)	13.6	3.6	2.7	2.7	0.6		
Problematic use of PA (>60 min a day) ^†^ (total) (%)	13.2	8	5.6	13.2	1.4	0.36	<0.0001
No > Low-Risk ED ED ^1^ (%)	12.2	6.8	4.5	9.2	0.7		
High-Risk ED ^2^ (%)	17.6	13.3	10	30	4.2		
Extreme weight loss (9 kg or more) * (total) (%)	10.3	–	–	–	–	0.21	<0.0001
No > Low-Risk ED ^1^ (%)	7.1 (Yes)	–	–	–	–		
High-Risk ED ^2^ (%)	23.6 (Yes)	–	–	–	–		

^†^ V—the Cramer’s V correlation coefficient; * φ—the Phi correlation coefficient; *p*—*p*-value, ^1^—EAT-26 score < 20, ^2^—EAT-26 score ≥ 20; PA—physical activity.

**Table 4:** Association between the EAT-26 scores, behaviours associated with ED and the self-reported BMI (underweight, overweight and obesity/normal weight).

Covariates (Nagelkerke R^2^ = 0.29)	Underweight (BMI < 18.5 kg/m^2^) ^a^				Overweight and Obese (BMI 25–34.9 kg/m^2^) ^a^			
	β	W	*p*	AOR (95% CI)	β	W	*p*	AOR (95% CI)
EAT-26 ^1^	−0.1	0.2	0.68	0.8 (0.5, 1.5)	0.6	9.5	0.002	1.8 (1.2, 2.7)
Factor D (dieting) ^2^	−0.7	12.1	0.004	0.5 (0.4, 0.8)	1.2	61.2	<0.0001	3.4 (2.5, 4.7)
Factor B (bulimia) ^3^	0.1	0.1	0.752	1.1 (0.8, 1.5)	0.1	0.1	0.784	1 (0.8, 1.4)
Factor O (oral control) ^4^	1.6	78.2	<0.0001	4.8 (3.4, 6.8)	−0.8	30.1	<0.0001	0.5 (0.3, 0.6)
Problematic use of PA (>60 min a day)	−0.2	9.8	0.002	0.8 (0.7, 0.9)	0.1	5.4	0.017	1.1 (1, 1.2)
Binge eating	−0.3	11.7	0.001	0.8 (0.6, 0.9)	0.1	5	0.027	1.1 (1, 1.3)
Self-induced vomiting	0.3	5.1	0.035	1.3 (1.1, 1.6)	−0.1	1.3	0.247	0.9 (0.7, 1.1)
Laxatives, diet pills, diuretics	0.3	5.3	0.036	1.3 (1, 1.7)	0.1	0.4	0.555	1.1 (0.8, 1.3)

^a^—reference category was normal weight (BMI 18.5–24.9); β—the estimated coefficient; W—the Wald test statistic; CI—confidence interval; *p*—*p*-value; BMI—body mass index; logistic regression model was adjusted for sex and branch of sciences; ^1^—EAT-26 scale score ≥ 20; ^2^—D (dieting) subscale score (≥3) is closely correlated with a distorted body image, ^3^—B (bulimia) subscale score (≥1) is closely associated to body weight and provides information about body image and tendency towards bulimic behaviour; ^4^—O (oral control) subscale score (≥2) is related to low body weight and to the absence of bulimia; PA—physical activity.

## Data Availability

Data are available on request.
